# The coronavirus nsp14 exoribonuclease interface with the cofactor nsp10 is essential for efficient virus replication and enzymatic activity

**DOI:** 10.1128/jvi.01708-24

**Published:** 2025-01-10

**Authors:** Samantha L. Grimes, Brook E. Heaton, Mackenzie L. Anderson, Katie Burke, Laura Stevens, Xiaotao Lu, Nicholas S. Heaton, Mark R. Denison, Jordan Anderson-Daniels

**Affiliations:** 1 Department of Pathology, Microbiology, and Immunology, Vanderbilt University Medical Center 204907 , Nashville, Tennessee, USA; 2 Department of Molecular Genetics and Microbiology, Duke University School of Medicine 12277 , Durham, North Carolina, USA; 3 Department of Pediatrics, Vanderbilt University Medical Center 12328 , Nashville, Tennessee, USA; Loyola University Chicago - Health Sciences Campus, Maywood, Illinois, USA

**Keywords:** coronavirus, exoribonuclease, replication, nsp14, nsp10, competitive fitness

## Abstract

Coronaviruses (CoVs) encode non-structural proteins (nsp’s) 1–16, which assemble to form replication-transcription complexes that function in viral RNA synthesis. All CoVs encode a proofreading 3′−5′ exoribonuclease in non-structural protein 14 (nsp14-ExoN) that mediates proofreading and high-fidelity replication and is critical for other roles in replication and pathogenesis. The *in vitro* enzymatic activity of nsp14-ExoN is enhanced in the presence of the cofactor nsp10. We introduced alanine substitutions in nsp14 of murine hepatitis virus (MHV) at the nsp14-nsp10 interface and recovered mutant viruses with a range of impairments in replication and *in vitro* biochemical exonuclease activity. Two of these substitutions, nsp14 K7A and D8A, had impairments intermediate between wild type-MHV nsp14 and the known ExoN(-) D89A/E91A nsp14 catalytic inactivation mutant. All introduced nsp14-nsp10 interface alanine substitutions impaired *in vitro* exonuclease activity. Passage of the K7A and D8A mutant viruses selected second-site non-synonymous mutations in nsp14 associated with improved mutant virus replication and exonuclease activity. These results confirm the essential role of the nsp14-nsp10 interaction for efficient enzymatic activity and virus replication, identify proximal and long-distance determinants of nsp14-nsp10 interaction, and support targeting the nsp14-nsp10 interface for viral inhibition and attenuation.

**IMPORTANCE:**

Coronavirus replication requires assembly of a replication transcription complex composed of nsp’s, including polymerase, helicase, exonuclease, capping enzymes, and non-enzymatic cofactors. The coronavirus nsp14 exoribonuclease mediates several functions in the viral life cycle including genomic and subgenomic RNA synthesis, RNA recombination, RNA proofreading and high-fidelity replication, and native resistance to many nucleoside analogs. The nsp-14 exonuclease activity *in vitro* requires the non-enzymatic cofactor nsp10, but the determinants and importance of the nsp14-nsp10 interactions during viral replication have not been defined. Here we show that for the coronavirus murine hepatitis virus, nsp14 residues at the nsp14-nsp10 interface are essential for efficient viral replication and *in vitro* exonuclease activity. These results shed new light on the requirements for protein interactions within the coronavirus replication transcription complex, and they may reveal novel non-active-site targets for virus inhibition and attenuation.

## INTRODUCTION

Coronaviruses (CoVs) cause endemic, epidemic, and pandemic diseases in humans and animals and demonstrate frequent cross-species movement and zoonotic diseases. Thus, determining fundamental mechanisms of replication is critical to understanding their evolution and targeting for inhibition and attenuation. CoVs are members of the order *Nidovirales* and possess the largest known and most complex single-stranded (+)RNA genomes of viruses known to infect humans and animals (1, 2). CoVs have significantly lower basal mutation rates than other RNA viruses. This relative higher fidelity of replication is mediated by the proofreading exoribonuclease (3′−5′ exoribonuclease [ExoN]), which is conserved in the large nidoviruses (3, 4). ExoN-mediated proofreading has been proposed to be a key factor in the ability of large nidoviruses to faithfully maintain genetic integrity of large genomes in the setting of selective pressures (3, 5).

**Fig 1:**
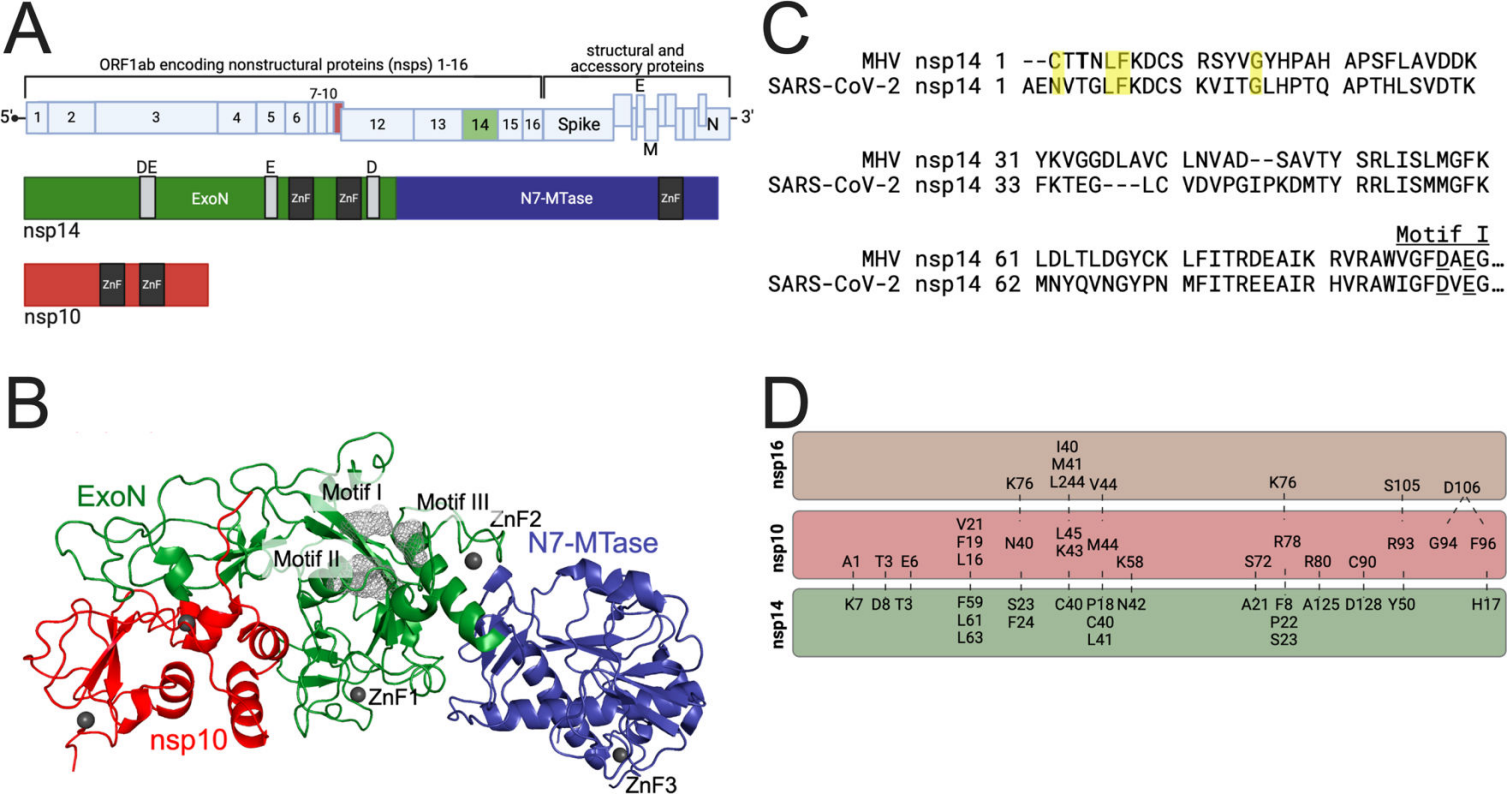
Coronavirus genome, non-structural protein 14 (nsp14) structure, conservation, and interface with nsp10. (**A**) Schematic of the severe acute respiratory syndrome coronavirus 2 (SARS-CoV-2) genome showing open reading frame (ORF) 1ab, which encodes non-structural proteins 1–16, as well as the region of the genome encoding structural and accessory proteins. Colored are nsp10 (red) and nsp14 (green/blue) (created with BioRender). Below, a linear depiction of the SARS-CoV-2 nsp14 domains showing the 3’−5’ exoribonuclease (ExoN) (green) and N7 methyltransferase (N7-MTase) (blue) domains. The ExoN catalytic motifs for SARS-CoV-2 are shown in light gray (motif I: D90 and E92, motif II: E191, motif III: D273). The zinc fingers are shown in dark gray. Shown also is nsp10, a cofactor for both nsp14 and nsp16 with the two zinc fingers shown in dark gray. (**B**) The cryogenic electron microscopy structure of the SARS-CoV-2 nsp14 bound to nsp10 (PDB: 7N0B). Colors are the same as in panel A, with domains and motifs labeled. (**C**) Amino acid alignment between the murine hepatitis virus (MHV) nsp14 (YP_009924354.1) and SARS-CoV-2 (YP_009724389), with residues of interest highlighted in yellow. (**D**) Diagram of the predicted nsp10-nsp14 interface and the nsp10-nsp16 interface in MHV. Dashed lines signify likely interactions based on biochemical and structural studies.

The 5′ two-thirds of the CoV (+)RNA genome encodes 16 non-structural proteins (nsp1–16) (Fig. 1) (6). Coronavirus replication in cells is initiated by the translation of the input (+)RNA genome into two coamino-terminal polyproteins encoding nsp1–16, followed by proteolytic maturation processing by two or three protease activities within the translated polyproteins (7–9). Structural, biochemical, and genetic studies have shown or predicted roles for multiple nsp’s in the formation and function of the membrane-associated replication-transcription complexes (RTCs) that mediate all stages of viral RNA synthesis (6). These include nsp12 RNA-dependent RNA polymerase (nsp12-RdRp) and its cofactors nsp7 and nsp8, nsp13 helicase-ATPase involved in RNA unwinding, nsp14 exonuclease and N7-methyltransfersase activities, nsp15 endoribonuclease, and nsp16 2′-O methyltransferase (6, 10–13).

The CoV nsp14 has two distinct functional domains, the amino-terminal 3′−5′ exoribonuclease (nsp14-ExoN) and the carboxy-terminal N7 methyltransferase (nsp14-N7-MTase) (Fig. 1). The nsp14-ExoN has many functions during CoV replication and pathogenesis. The exonuclease activity of ExoN is dependent on four conserved active residues in three motifs: motif I-DE, motif II-E, and motif III-DH. The nsp14 exonuclease hydrolyses ssRNA and dsRNA in a 3′–5′ direction (14) and excises 3′ single-nucleotide mismatches in template RNA (15). ExoN is required for wild-type (WT) viral RNA accumulation and subgenomic RNA synthesis (14). Viable mutants of SARS-CoV and murine hepatitis virus (MHV) with alanine substitutions at ExoN motif I (DE) residues (hereafter ExoN(−)) have (i) defects in virus replication and RNA synthesis (3, 16); (ii) up to 20-fold increased mutation frequency compared to WT parental viruses and more consistent with higher mutation rates of other RNA viruses (3, 16); (iii) increased sensitivity to nucleoside analogs including 5-fluorouracil (5-FU), remdesivir, and molnupiravir (5, 17, 18); (iv) decreased and altered recombination functions during genomic and subgenomic RNA syntheses (19); (v) increased sensitivity to interferon (20, 21); (vi) loss of fitness compared to WT virus (22); and (vii) *in vivo* attenuation of an ExoN(−) mutant of lethal mouse-adapted SARS-CoV (23). More recent studies in porcine epidemic diarrhea virus, SARS-CoV, and severe acute respiratory syndrome coronavirus 2 (SARS-CoV-2) support the role of nsp14 as an innate immune antagonist (20, 24, 25).

Studies of nsp14 structure and exonuclease activity have demonstrated that nsp10, a small non-enzymatic protein, has specific interactions with nsp14 (26–33). The *in vitro* exonuclease activity of nsp14-ExoN requires or is enhanced by nsp10, as is stability and resolution of nsp14 structures solved by crystallography or cryogenic electron microscopy (Fig. 1) (27–31, 33, 34). Nsp10 also is a required cofactor for the *in vitro* activity and stable structure of nsp16 2′-O-methyltransferase (35). The residues of nsp10 that interact at an interface with nsp14 share a partially overlapping footprint with the residues that interact with nsp16-2′-OMTase (Fig. 1) (28). Amino acid substitutions at nsp10 residues that interface with nsp14 limit nsp10 association with nsp14, impair nsp14-exonuclease activity *in vitro*, and may impact N7-MTase activity (36). In our genetic studies with MHV, alanine substitutions in nsp10 at some residues proposed to interface with nsp14 were non-viable, while others were viable but had altered sensitivity to nucleoside analogs under different thermal conditions (37). However, genetic and biochemical studies of mutations in nsp10 are limited for conclusions about impact of the nsp14-nsp10 interface because of our evolving but limited understanding of the structural and biochemical interactions of nsp10, nsp14, and nsp16 during virus replication; interactions in the larger RTC during viral RNA synthesis; and impact on other nsp14 functions. A recent study reports a heterotrimeric structure of SARS-CoV-2 nsp10-14-16 (38). However, during infection, assembly of CoV RTC proteins, and genome replication, it is not known if nsp10 molecules shuttle or compete between nsp14 and nsp16, or if distinct nsp10 molecules exclusively interact with one protein or the other. Therefore, understanding the role of the nsp14-nsp10 interface during infection requires genetic and biochemical approaches targeting nsp14.

In this study, we show that alanine substitutions in MHV nsp14 residues at the nsp14-nsp10 interface variably impact virus replication, exonuclease activity, and nucleoside analog sensitivity. Two nsp14 substitutions, K7A and D8A, had the greatest impact on replication, exonuclease activity, and fitness but did not increase sensitivity to nucleoside analogs. Passage of these mutants partially restored virus replication but remained less fit than WT MHV and selected for second-site substitutions in nsp14 that, when reintroduced into the mutant virus background, independently partially compensated replication and biochemical activities. Together these data demonstrate that the nsp14-nsp10 interface, and specifically the nsp14 residues at this interface, are key regulators of nsp14 functions but have functions in addition to facilitating exonuclease activity.

## RESULTS

**Fig 2:**
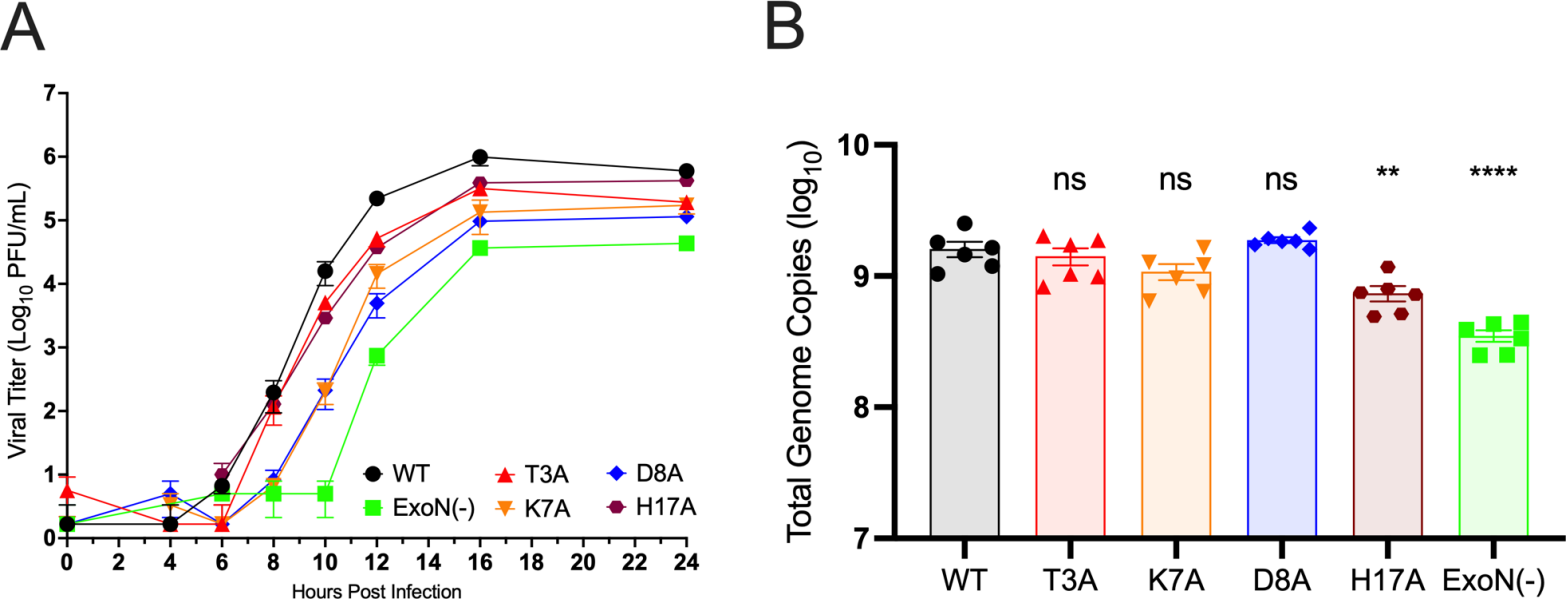
Replication of nsp14–nsp10 interface mutants. Replication of MHV-WT, ExoN(−), nsp14-T3A, nsp14-K7A, nsp14-D8A, and nsp14-H17A. (**A**) DBT-9 cells were infected with the indicated virus at an MOI of 0.01 PFU/cell. Supernatant samples were collected at the indicated times, and the virus titer was determined by plaque assay. Data shown are from three independent experiments, each with three replicates. Points represent experiment means (*n* = 3) ± SEM. (**B**) DBT-9 cells were infected with the indicated viruses at an MOI of 0.5 PFU/cell. Monolayers were harvested in TRIzol at 8 h post infection. RNA was purified, and total viral genomes were quantified by reverse transcription quantitative PCR. Graphed are the individual means from duplicate infections across three independent experiments (*n* = 6) ± SEM. **P < 0.01, ****P < 0.0001. ns, not significant (determined by one-way analysis of variance with Dunnett’s multiple-comparison test).

We introduced mutations coding for alanine substitutions at four residues in the MHV nsp14 at the proposed nsp14-nsp10 interface that were conserved across divergent CoVs: T3, K7, D8, and H17 (Fig. 1). Using reverse genetics, we recovered MHV harboring the intended mutations and generated low-passage stocks that were used for all experiments presented. Both T3A and H17A mutant viruses produced WT-like plaques in delayed brain tumor clone 9 (DBT-9) cells, while K7A and D8A mutant viruses produced small and medium-size plaques. We compared the replication kinetics of the mutant viruses to that of WT MHV and to the well-characterized, catalytically inactive nsp14-ExoN(−) mutant (D89A/E91A) (Fig. 2). T3A and H17A viruses began replication with similar kinetics as WT MHV; however, both mutant viruses had peak titers less than WT-MHV. In contrast, the K7A and D8A viruses demonstrated a 2 h delay to onset of exponential replication compared to WT and had impaired peak titers 10- to 50-fold less than WT-MHV. All four mutant viruses demonstrated earlier onset of exponential replication and increased peak titers compared to nsp14-ExoN(−). To determine if the impact on infectious particle production correlated with RNA synthesis, we infected DBT-9 cells with the nsp14-nsp10 interface mutants at an MOI of 0.5 PFU/cell and quantified cell-associated viral RNA after a single round of infection at 8 h post infection. The ExoN(−) mutant virus had a significant decrease in RNA production compared to WT MHV (Fig. 2), consistent with our previous reports (39). The T3A, K7A, and D8A mutant viruses produced RNA at WT levels, while H17A had a small but statistically significant decrease in RNA synthesis compared to WT MHV. These data indicate that nsp14 residues at the nsp10 interface contribute to efficient MHV replication and infectious particle production.

### Competitive fitness of mutant viruses

**Fig 3:**
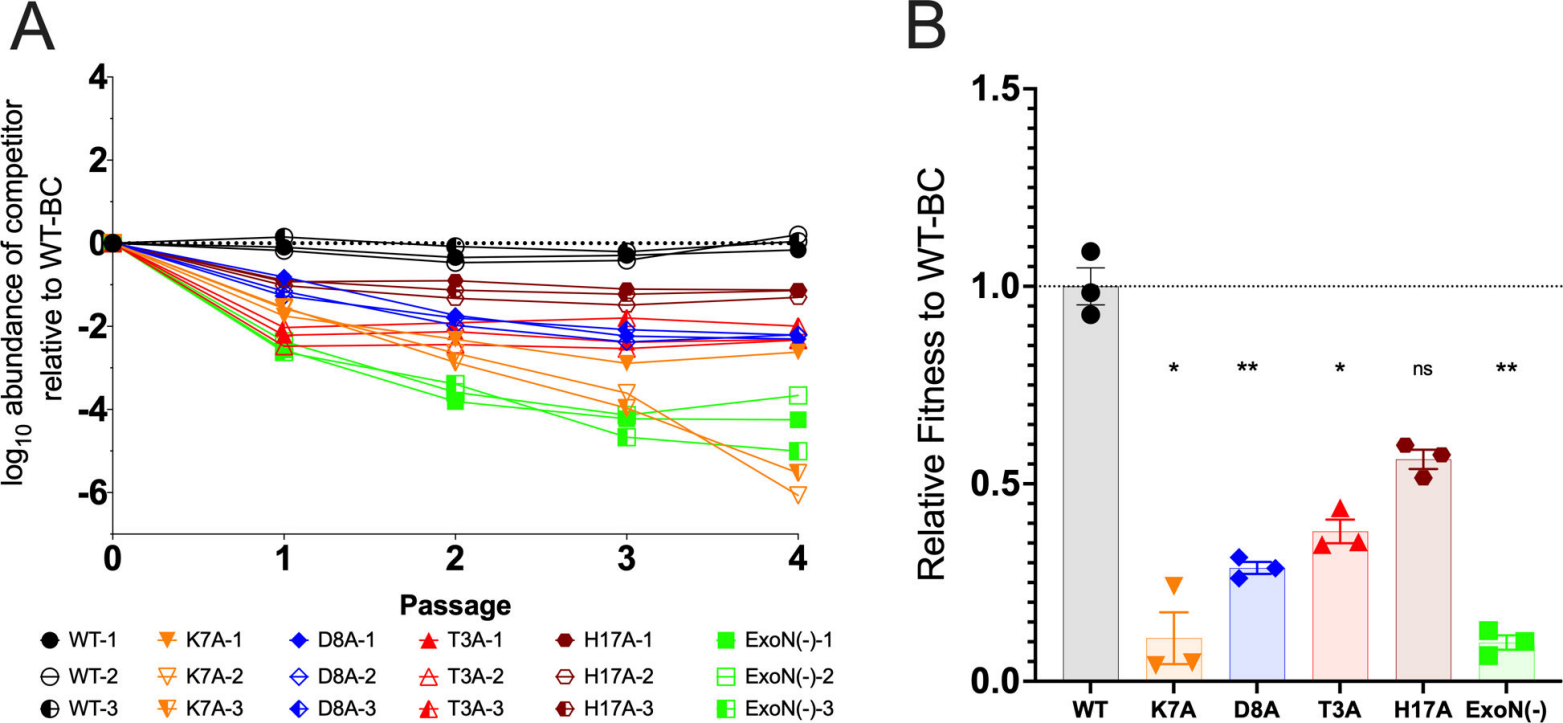
Competitive fitness of nsp14-nsp10 interface mutants. (**A**) DBT-9 cells were coinfected with a barcoded WT MHV and a non-barcoded WT, K7A, D8A, T3A, H17A, or ExoN(−) at a combined MOI of 0.1 PFU/cell. The resulting supernatants were passaged four times. The relative quantities of barcoded and non-barcoded cDNAs were plotted over passage for the three independent lineages of each competition, normalized to the input amounts. (**B**) Linear regression from panel A was used to determine the relative fitness for each non-barcoded virus. Individual data means are graphed (*n* = 3) ± SEM. *P < 0.05, **P < 0.01. ns, not significant (determined by one-way analysis of variance with Dunnett’s multiple-comparison test).

We next tested the fitness of T3A, K7A, D8A, and H17A viruses using a coinfection competitive fitness assay (39–41) (Fig. 3). Three independent lineages of mutant and WT viruses were coinfected with competitor WT MHV encoding a genetic barcode of seven silent mutations in the nsp2 coding domain (WT-BC). The resulting viral populations were then passaged an additional three times at a constant MOI of 0.1 PFU/cell. Viral population RNA was extracted from the supernatants of each passage, and primers detecting either the barcoded (WT-BC infection) or non-barcoded (WT control, mutant competitor) nsp2 cDNA were used in reverse transcription quantitative PCR (RT-qPCR) reactions. The ratio of non-barcoded to barcoded cDNA was plotted over passage number. All mutant virus cDNAs were less abundant than WT by passage 1 (P1) with varying degrees of downward trends through passage 4. We analyzed the competitor:WT-BC ratio by linear regression to determine the competitive fitness of each mutant relative to WT MHV. T3A, K7A, and D8A mutant competitors had significant reductions in fitness relative to WT, while the fitness of H17A was not significantly different from WT. These data are consistent with the replication kinetics data.

### Mutant virus sensitivity to nucleoside analogs

**Fig 4:**
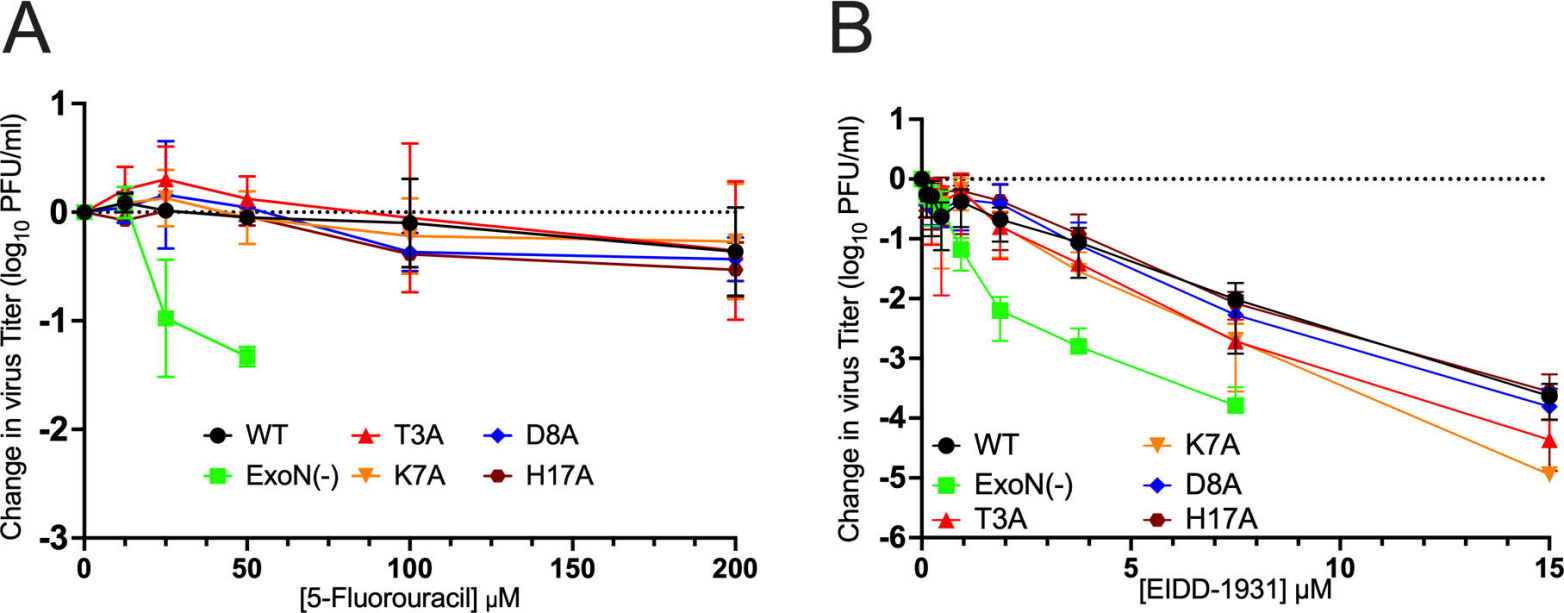
Nucleoside analog sensitivity of nsp14-nsp10 interface mutants. (**A**) Change in viral titer in response to varying concentrations of the nucleoside analog 5-fluorouracil. DBT-9 cells were infected with the indicated virus at an MOI of 0.01 PFU/cell and treated with the indicated concentration of 5-fluorouracil. Supernatant samples were collected at 24 hpi, and virus titer was determined by plaque assay. Points represent experimental means (*n* = 2) ± SEM. (**B**) Change in viral titer in response to varying concentrations of the active form of the nucleoside analog molnupiravir, EIDD-1931, for the indicated viruses. DBT-9 cells were infected with the indicated virus at an MOI of 0.01 PFU/cell and treated with the indicated concentration of EIDD-1931. Supernatant samples were collected at 24 hpi, and virus titer was determined by plaque assay. Points represent experiment means (*n* = 3) ± SEM.

WT MHV has native resistance to several nucleoside analogs, a trait that is associated with intact nsp14 catalytic activity. The MHV-ExoN(−) mutant virus has increased inhibition by multiple nucleoside analogs, including 5-FU, consistent with loss of proofreading or enhanced selectivity with loss of ExoN function (5). To determine if the nsp14-nsp10 interface mutants share this phenotype with MHV-ExoN(−), we compared the 5-FU sensitivities of WT-MHV, MHV-ExoN(−), and the T3A, K7A, D8A, and H17A mutant viruses (Fig. 4). WT-MHV was unaffected by 5-FU concentrations up to 200 µM, while MHV-ExoN(−) was inhibited in a dose-dependent manner with complete inhibition by 100 µM 5-FU. In contrast, the T3A, K7A, D8A, and H17A viruses all retained WT-like resistance to 5-FU. We next tested mutant sensitivity to the nucleoside analog EIDD-1931, a cytidine analog and active form of the antiviral molnupiravir with broad-spectrum activity against CoVs and which functions as an RNA mutagen during replication. Consistent with previous experiments, WT MHV demonstrated dose-dependent inhibition by EIDD-1931, and MHV-ExoN(−) had enhanced sensitivity to EIDD-1931 at all concentrations tested up to 15 µM (18). In contrast, the T3A, K7A, D8A, and H17A mutant viruses retained WT-like inhibition by EIDD-1931 up to 2 µM, with increases in inhibition between 2 and 15 µM. They were 100-fold less sensitive to EIDD-1931 at 2 µM compared to MHV-ExoN(−), and mutant virus infection was detectable at all EIDD-1931 concentrations tested. Thus, the interface substitutions did not directly impact the sensitivity to either 5-FU or EIDD-1931.

### Biochemical exonuclease activity of nsp14-nsp10 interface mutants

**Fig 5:**
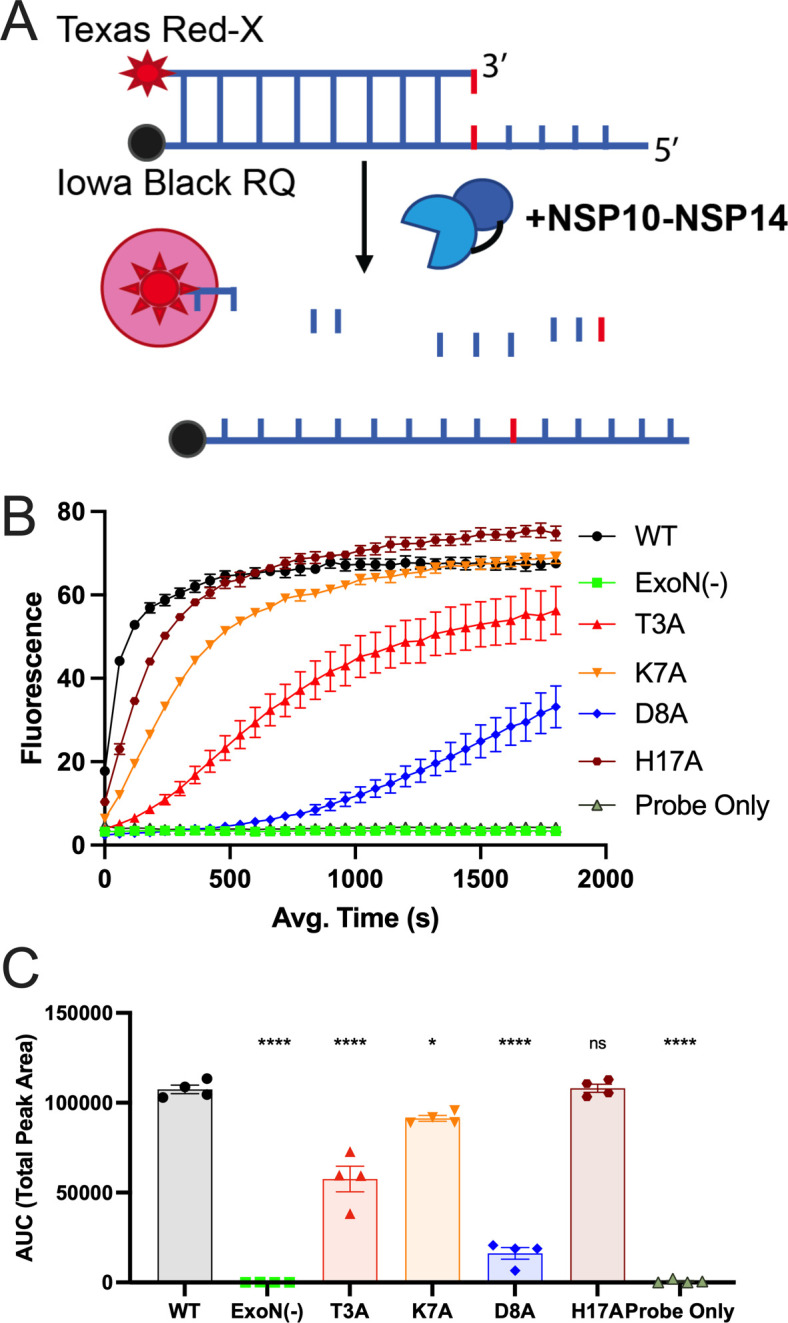
RNA cleavage of nsp14-nsp10 interface mutants. (**A**) The *in vitro* exonuclease activity was determined by a fluorescence RNA cleavage assay using nsp10/nsp14 fusion proteins encoding interface substitutions. The Texas red fluorescence increases with increased exonuclease activity on the partially double stranded (DS) RNA template. (**B**) The RNA cleavage activity of WT MHV nsp14, nsp14-ExoN(−), and interface mutants was measured over time using normalized amounts of protein and 250 nM probe. Points represent experiment means (*n* = 4) ± SEM. (**C**) Area under the curve analysis for the first 500 s of the reactions shown in panel **B**. Points represent experiment means (*n* = 4) ± SEM. *P < 0.05, ****P < 0.0001. ns, not significant (determined by one-way analysis of variance with Dunnett’s multiple-comparison test).

To test the exonuclease activity of nsp14 T3A, K7A, D8A, and H17A mutants, we performed *in vitro* exonuclease activity experiments using a recombinant MHV nsp10/nsp14 fusion protein biochemical assay adapted from (42) (Fig. 5). WT and mutant MHV nsp10/nsp14 proteins were expressed, purified, and incubated with a double-stranded RNA molecule containing a 5′-TexasRed fluorophore and 3′-quencher. Exonuclease activity was quantified by fluorescence readout over time with increasing signal indicating increased exonuclease activity. The WT MHV nsp10/nsp14 fusion protein generated robust signal, while the MHV-ExoN(−) mutant (D89A/E91A) protein signal was indistinguishable from probe alone, confirming the complete biochemical inactivation of nsp14-ExoN. (Fig. 5). The T3A, K7A, D8A, and H17A mutants each demonstrated retention or impairment of exonuclease activity, consistent with the impact of mutations on replication and fitness, but did not correlate with the retention of native resistance to nucleoside analogs 5-FU and EIDD-1931. To further compare the exonuclease activities of each mutant to the WT nsp10/nsp14 protein, we calculated the area under curve for each protein construct prior to WT saturation at 500 s. With the exception of H17A, each mutant protein had significantly reduced fluorescence during this time course compared to WT. These data indicate that nsp14 mutations at the nsp10 interface can impair nsp14 exonuclease *in vitro* activity to varying degrees but also suggest that exonuclease impairments may be decoupled from viral sensitivity to mutagenic nucleoside analogs.

### Passage adaptation of nsp14 interface mutants K7A and D8A

**Fig 6:**
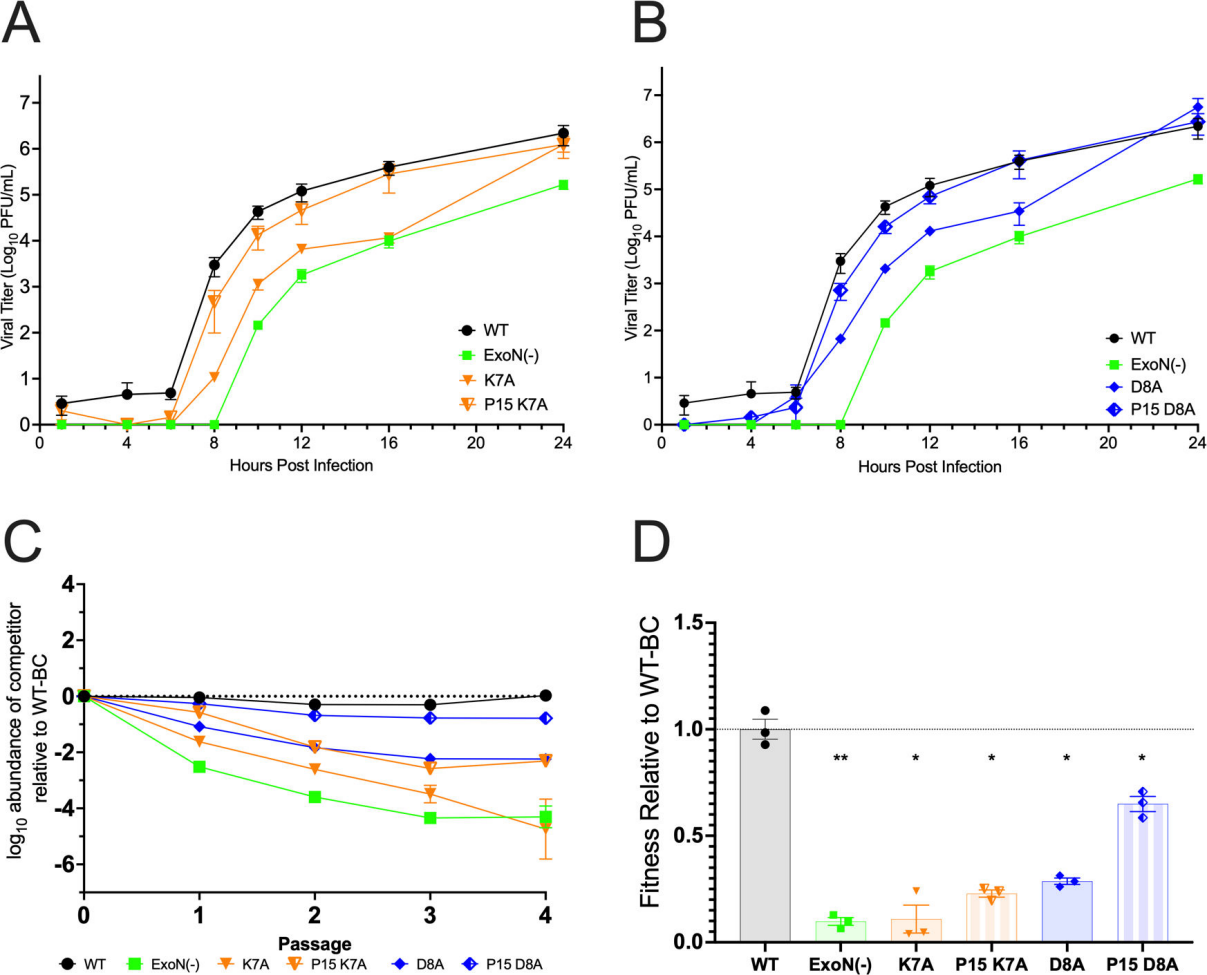
Replication and fitness of nsp14-nsp10 interface mutant passage populations. (**A and B**) DBT-9 cells were infected with the indicated virus at an MOI of 0.01 PFU/cell. Supernatant samples were collected at the indicated times, and the virus titer was determined by plaque assay. Data shown are from three independent experiments, each with three replicates. Points represent experiment means (*n* = 3) ± SEM. Replication of K7A P1 and P15 (**A**) and D8A P1 and P15 (**B**) compared to WT and ExoN(−). (**C**) Competitive fitness of nsp14 mutant passage population viruses. Graphed are mean values from three independent lineages. (**D**) Linear regression from panel C was used to determine the relative fitness for each non-barcoded virus. Individual data means are graphed (*n* = 3) ± SEM. *P < 0.05, **P < 0.01 (determined by one-way analysis of variance with Dunnett’s multiple-comparison test).

Based on the significant impairment of D8A and K7A mutant replication and exonuclease activities, we passaged these viruses to identify potential intra- and intermolecular interactions of interface residues (Fig. 6). Starting with the P0 viral stocks, we serially passaged WT MHV alongside K7A and D8A mutant viruses in DBT-9 cells. Infected cell supernatants were passaged 15 times, by which all virus populations demonstrated similar cytopathic effect (CPE) (syncytia formation and cell loss) at the time of harvesting. We then compared the replication kinetics and competitive fitness of mutant passage population viruses at P1 and passage 15 (P15) to WT MHV (Fig. 6). For both the K7A and D8A mutants, passaging selected for a more rapid onset of exponential replication so that by P15, both populations demonstrated replication kinetics and peak titers closer to or indistinguishable from WT MHV. Both D8A and K7A P15 populations had higher relative fitness compared to the P1 viruses but remained less fit compared to WT.

### Sequence analysis of passage populations

We performed RNA sequencing (RNA-seq) of the P1 and P15 virus populations to identify minority variants and other mutations across the genome that arose during passaging (Table 1). Passage of WT-MHV showed low-level tissue culture adaptive changes in E and M structural proteins but no detectable coding changes with frequencies above 0.01 in any replicase non-structural protein. The K7A mutant also had low level (<0.12) aa substitutions in E and S, and the D8A mutant had a single substitution in ORF4a at a frequency of 0.17. Both K7A and D8A retained their primary introduced changes and selected for other substitutions in nsp14. The K7A mutant passage selected an nsp14 V82I substitution (P1-0.23 and P15-0.76) as well as P15 substitutions of >0.1 in nsp1, nsp3, and nsp8. The D8A mutant passage selected for three substitutions in nsp14: same-site substitution D8V (P15-0.23), T49N (P1-0.07 and P15-0.41), and R52Q (P1-0.18 and P15-0.12), as well as P15 substitutions of >0.1 in nsp12 and nsp13. Because all three adaptive nsp14 substitutions were present at P1 in K7A and D8A, we performed Sanger sequencing of P0 recovered virus from infected cell lysates, which showed a minor peak encoding V82I along with V82 in the K7A P0. In contrast, theT49N and R52Q substitution mutations were not observed at P0. Sanger sequencing confirmed that the V82I mutation was not present in the cloned K7A cDNA fragment used to generate the virus. These results are the first to show selection of mutations in nsp14 proximate and more distant from the interface, and their early appearance suggests they may have been necessary for efficient growth of the K7A and D8A viruses during recovery. This significant selective pressure was also supported by the appearance of the same site D8V substitution which was not detected at P0 or P1 but emerged by P15.

**TABLE 1:** RNA-seq analysis of K7A and D8A passage populations^*b*^

K7A			
Protein	aa substitution	Frequency P1	Frequency P15
nsp1	S210C	0.219	0.562
nsp3	K20R	0.036	0.116
nsp8	A75S	0.232	0.670
**nsp14**	**K7A**	**0.995**	**0.995**
**nsp14**	**V82I**	**0.228**	**0.758**

D8A			
Protein	aa substitution	Frequency P1	Frequency P15
nsp12	G810S	0.209	0.139
nsp13	R129G	0.018	0.193
**nsp14**	**D8A**	**0.770**	**0.770**
**nsp14**	**D8V**	**ND**	**0.230**
**nsp14**	**T49N**	**0.072**	**0.410**
**nsp14**	**R52Q**	**0.179**	**0.122**

^*a*^ The nsp14 mutants were serially passaged 15 times. DBT-9 cells were infected with the indicated population virus, and RNA was extracted from infected cell monolayers and sequenced. Coding mutations across the genome with >0.1 frequency are reported, showing the resultant protein substitution and frequency.

^*b*^ Boldface indicates to serve as quick reference for the reader.

### Replication of K7A and D8A with engineered nsp14 substitutions

**Fig 7:**
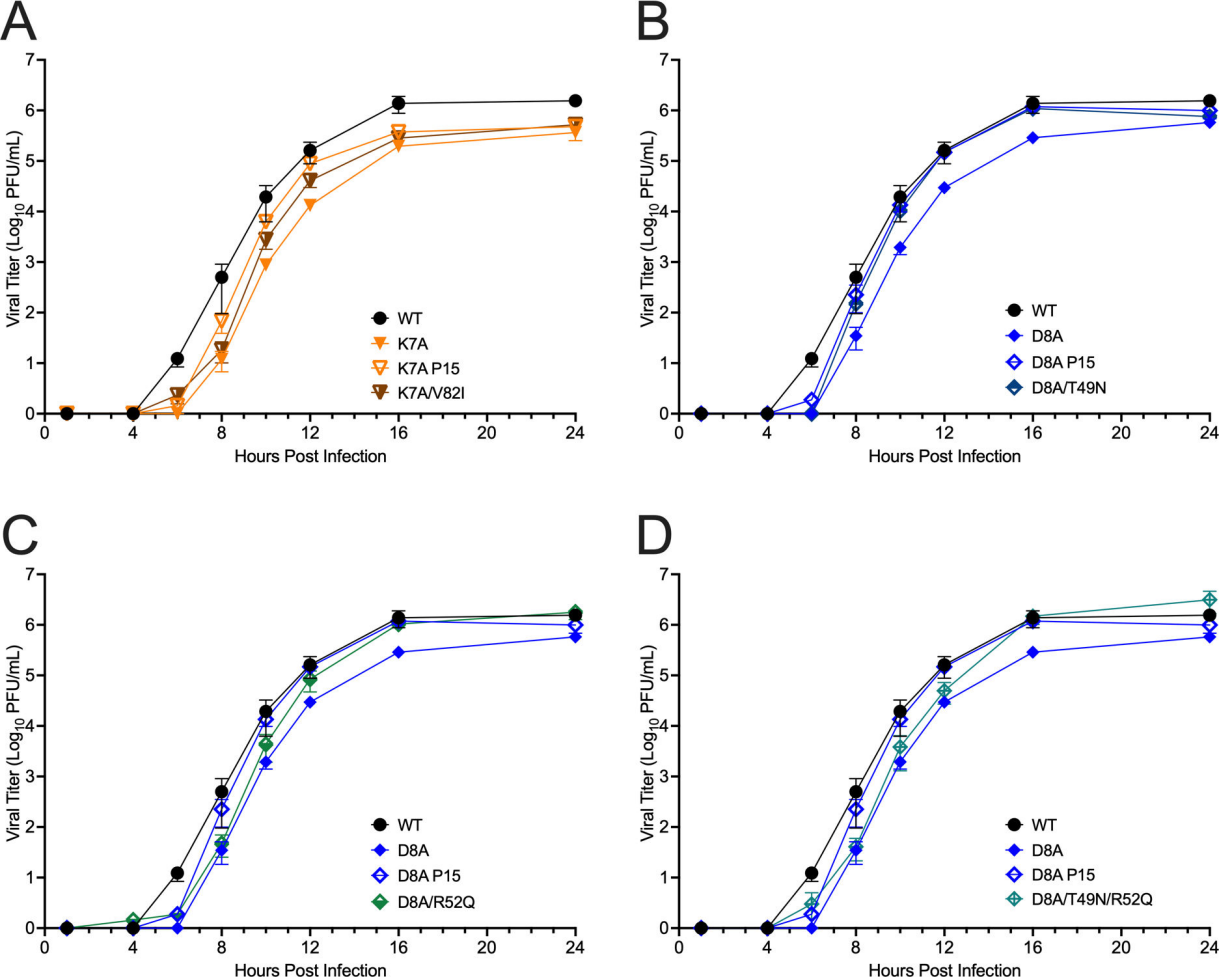
Replication of engineered nsp14-nsp10 interface mutants. Replication of WT MHV and mutant viruses. DBT-9 cells were infected with the indicated virus at an MOI of 0.01 PFU/cell. Supernatant samples were collected at the indicated times, and the virus titer was determined by plaque assay. Data shown are from three independent experiments, each with three replicates; data are separated into four panels for ease of viewing. Points represent experiment means (*n* = 3) ± SEM. (**A**) K7A, K7A-P15, and K7A-V82I. (**B**) D8A, D8A-P15, and D8A-T49N. (**C**) D8A, D8A-P15, and D8A-R52Q. (**D**) D8A, D8A-P15, and D8A-T49N-R52Q.

We tested the contribution of the passage-selected nsp14 substitutions on replication by engineering the changes in the presence of K7A or D8A and recovering mutant viruses. We compared WT-MHV to the original engineered mutations (K7A and D8A), their P15 populations, and the viruses encoding K7A-V82I, D8A-T49N, D8A-R52Q, and D8A-T49N-R52Q (Fig. 7). The K7A-V82I virus had intermediate replication between K7A and the P15 K7A population. D8A-T49N, D8A-R52Q, and D8A-T49N-R52Q all had replication intermediate between D8A and D8A-P15 populations, with the exception that they achieved titers equal to D8-P15 and WT-MHV by late times of infection (16 hpi). For D8A, the results show that either T49N or R52Q alone is sufficient to improve the impaired replication kinetics of D8A, but there is no additive effect from both mutations in a D8A background. These data, paired with the frequency of T49N and R52Q, suggest that these substitutions may have been selected independently, and D8A-T49N-R52Q may not represent the genotype for individuals in the population. Overall, the experiments show that for both the K7A and D8A mutants, the selected mutations in nsp14 alone or together contribute to the adaptation of replication in isolation from other selected mutations or tissue culture adaptation.

### Exonuclease activity of nsp14 adaptive mutants

**Fig 8:**
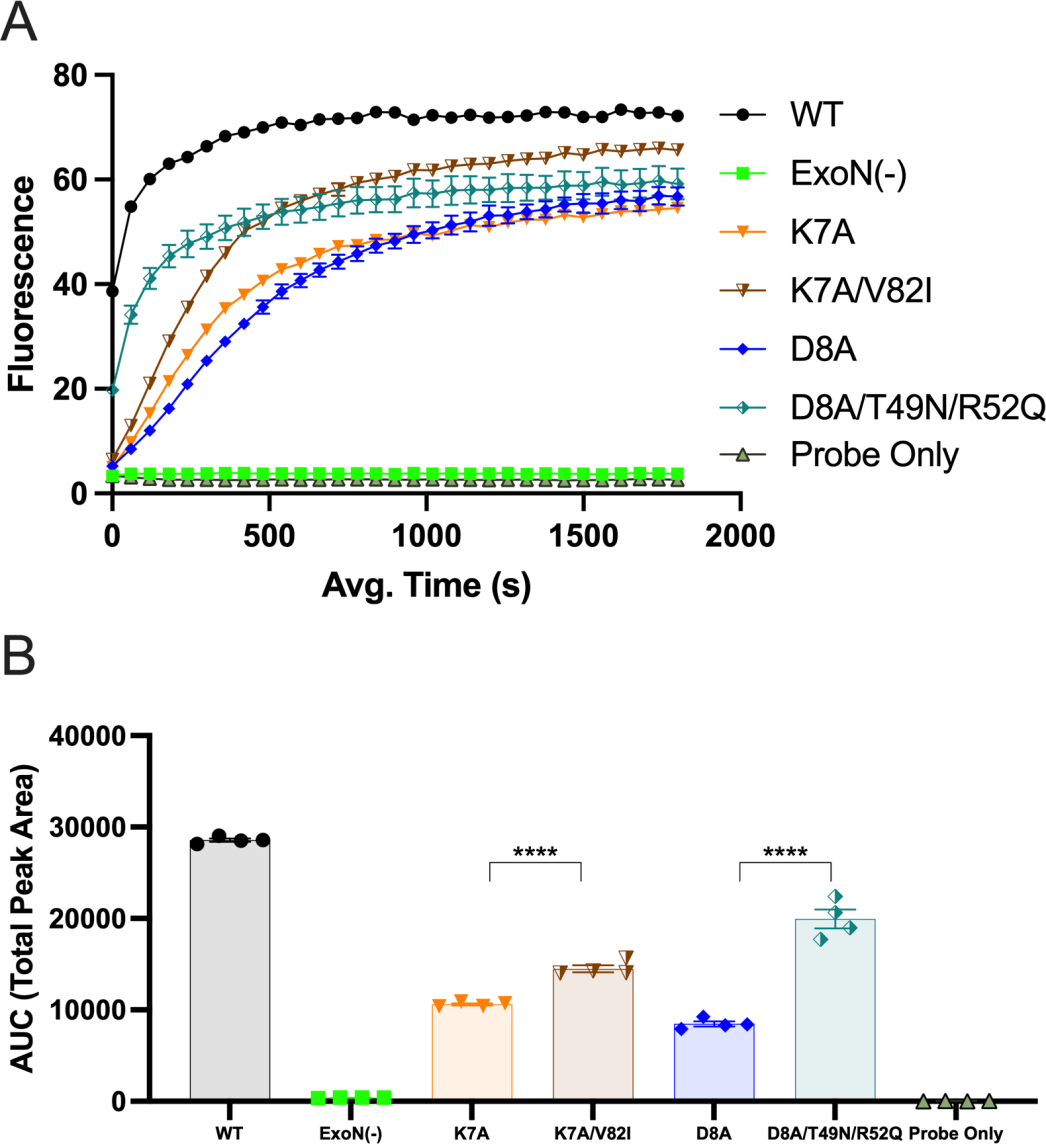
RNA cleavage of nsp14 adaptive mutants. (**A**) The *in vitro* exonuclease activity was determined by a fluorescence RNA cleavage assay using 500 nM WT protein or mutant nsp10/nsp14 fusion protein normalized to WT by SDS-PAGE mixed with 250 nM of probe. Points represent experiment means (*n* = 4) ± SEM. (**B**) Area under the curve analysis for the first 500 s of the reactions shown in panel **A**. Points represent experiment means (*n* = 4) ± SEM. ****P < 0.0001 (determined by unpaired *t*-test).

Finally, we determined the impact of the nsp14 passage-adaptive mutants on the exonuclease activity of nsp14. We generated nsp10/nsp14 fusion constructs with K7A-V82I substitutions and D8A-T49N-R52Q substitutions and compared the exonuclease activity to K7A, D8A, and WT under the same conditions listed above (Fig. 8). The K7A-V82I and D8A-T49N-R52Q both had increases in total fluorescence compared to K7A and D8A, respectively, as well as significant increases in the area under the curve (AUC) at timepoints less than 500 s, indicating the ability of these residues to partially compensate for the defects in exonuclease activity of the K7A and D8A mutants; however, neither construct approached WT levels of exonuclease activity. These data suggest that passaging nsp14 interface mutant viruses can act as selective pressure to identify residues outside of the canonical catalytic domains that impact exonuclease activity and other functions of the nsp14-ExoN domain.

## DISCUSSION

Here we show that amino acid substitutions in the MHV nsp14 at the interface with nsp10 result in varying degrees of impairment of replication, fitness, and biochemical exonuclease activity. Solved structures of nsp14/nsp10 complexes and biochemical experiments implicate multiple contact residues between the two proteins (26–30). Our data demonstrate that individual nsp14 residue changes may not completely abolish the interactions of nsp14 and nsp10. Future experiments are needed to fully map the requirements of each interface residue, and it is possible that changes at other interface residues not tested here are absolutely required for protein-protein interaction and particle viability. For example, the same-site selection of D8V in the D8A P15 population suggests that other residues at the location may be tolerated and/or restore the defect. Nonetheless, these experiments contribute to the growing understanding of the complex protein-protein interactions responsible for coronavirus replication.

Many studies of CoV nsp14 biochemical and replication activities have focused on the exoribonuclease domain catalytic motifs and have shown nsp14 functions during RNA synthesis, genome replication fidelity, subgenomic mRNA transcription, and interferon antagonism (3, 5, 15, 16, 19, 26, 36, 40). The well-characterized MHV nsp14 catalytic motif I mutant virus D89A/E91A (ExoN(−)) and homologous SARS-CoV ExoN(−) mutant have been powerful tools for probing nsp14 and CoV RTC functions. Our results in this study indicate that targeting the nsp14-nsp10 interface can yield viral and biochemical phenotypes intermediate between WT and ExoN(−). Thus, genetic alterations at the nsp14-nsp10 interface may provide a novel approach for experiments aimed at understanding the functions of nsp14 in pathogenic CoVs.

We focused our investigation on the K7A and D8A mutant viruses because they had the largest impairment on replication kinetics. The lack of impairment to RNA synthesis was surprising, especially considering the structural and biochemical studies that implicate the homologous SARS-CoV-2 K9 residue with RNA binding (29, 30). While our biochemical exonuclease data for K7A agree with those previously reported for SARS-CoV-2 K9A (29), our RT-qPCR data (Fig. 2) suggest that this conserved lysine residue is not a critical determinant for RNA synthesis during viral replication, or at least not for MHV. This result is potentially confounded by the presence of V82I in the population. Since V82I was present at low levels in both P0 and P1 populations, it is possible that this mutation was critical for the recovery of K7A and potentially compensates for defects in RNA synthesis. However, it remains to be determined how much of the P0 and P1 populations harbor both K7A and V82I substitutions on the same molecule. Together, these data further differentiate the nsp14-nsp10 interface mutations from ExoN(−) phenotypes, indicating that targeting the nsp14-nsp10 interface is an alternative approach to understanding the function of nsp14 during infection.

**Fig 9:**
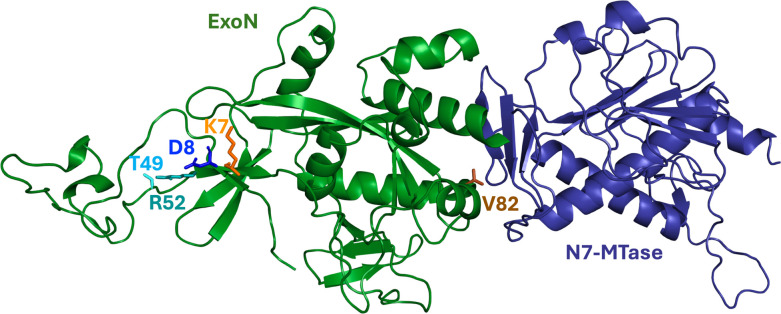
*De novo* model of MHV nsp14. PyMOL structure of the ColabFold *de novo* model of the MHV nsp14 showing the 3′−5′ exoribonuclease (ExoN) (green) and N7 methyltransferase (N7-MTase) (blue) domains. The sites of alanine mutagenesis are shown by colored side chains K7A (orange) and D8A (blue). The residues with mutations identified by Sanger sequencing and RNA sequencing are also shown: T49 and R52 residues (D8A passage) are shown in cyan and teal. V82 residue (K7A passage) is shown in brown.

Our passage experiments of K7A and D8A mutant viruses were designed to add selective pressures for improved replication; 15 passages were sufficient for partial restoration of replication, exonuclease activity, and fitness. The resultant D8A-P15 population harbored T49N and R52Q coding changes, while the K7A-P15 population harbored a V82I coding change. Since the structure of MHV nsp14 has not been reported, we performed ColabFOLD *de novo* modeling of the MHV nsp14 based on the solved structure of the SARS-CoV-2 nsp14 (PDB: 7N0B) (Fig. 9). Structural alignment of MHV and SARS-CoV-2 nsp14 (RMSD of 1.36) indicate that T49 and R52 residues are located proximal to the D8 residue near other residues reported to interact with nsp10. In contrast, the V82 residue is located distal from the nsp10 interface near the N7-MTase domain of nsp14. The results suggest that there are both close- and long-range communication nodes in the ExoN domain that determine its function and that multiple intra- or intermolecular adaptive pathways are possible with nsp14-nsp10 interface mutations, and they suggest that the CoV RTCs may adopt multiple conformations and protein-protein interactions during replication. This is supported by our recent report showing that viable mutants of MHV can be recovered with deletions of the cleavage sites at the amino and carboxy termini of nsp14, resulting in nsp13-14 and nsp14-15 obligate fusion proteins in the CoV RTC (39).

Our previous studies with MHV-ExoN(−) mutant viruses demonstrated a correlation between exonuclease activity and native resistance to mutagenic nucleoside analogs such as 5-FU (5). Experiments in this study demonstrate that biochemical exonuclease activity of nsp14-nsp10 interface mutants could be significantly impaired while retaining WT-like resistance to mutagenic nucleoside analogs 5-FU and EIDD-1931. This decoupling of exonuclease activity and mutagen sensitivity was thus unexpected and raises important questions about the role of nsp14-nsp10 interactions in the replication-transcription complex in relation to nucleotide sensitivity and proofreading. Possibilities for investigation include changes in how nsp14 interacts with the nsp12-RdRp for selectivity or that nucleotide removal is only altered once the exonuclease activity is reduced below a threshold.

A biochemical assay using an nsp10/nsp14 fusion protein was previously developed using SARS-CoV-2 proteins, in part to help identify nsp14 inhibitors (42). The MHV nsp10/nsp14 fusion protein system and exoribonuclease assay described here is a powerful tool which can be used as a parallel model with MHV genetic and *in vivo* studies to compare enzymatic activity with viral activity during infection in a useful BSL-2 CoV model. This fusion protein can be adapted to examine functions such as exoribonuclease activity on WT RNAs, RNA with incorporated nucleoside analogs, or in the context of nsp10 or nsp14 inhibitors. Furthermore, this MHV nsp10/nsp14 fusion protein may serve as the basis for a buildable *in vitro* model of the CoV RTC by incorporating multiple replication nsp’s or using additional approaches like magnetic tweezers for real-time analysis of the role of nsp14-nsp10 with multiple other replicase proteins during RNA synthesis, capping, and proofreading (43).

While this study provides important new insights, there are limitations and questions that are not addressable by this system of fused nsp10/nsp14 proteins. Recent structural studies have demonstrated dynamic arrangements of the SARS-CoV-2 nsp14 N-terminal domain when nsp10 is absent (31, 33) or in combination with nsp10 and nsp16 (38) compared to solved structures of nsp10-nsp14. It is currently unclear if the nsp10/nsp14 fusion protein system recapitulates the structural interactions or stoichiometric ratios of nsp10 and nsp14 present during CoV replication and does not account for the potential for competition or cooperation with nsp12, nsp16, or the other components of the replicase. Additionally, it is unclear if the exonuclease activity of the nsp10/nsp14 protein and by extension the impairment observed by the mutant proteins are the result of *cis* or *trans* interactions.

Together, these results demonstrate for the first time the contribution of nsp14 residues to the nsp14-nsp10 interface and importance in virus replication and fitness, identify the interface as a compelling target for antiviral drugs, and introduce a novel tool for parallel genetic and biochemical analyses in a model CoV.

## MATERIALS AND METHODS

### Cell culture

Murine astrocytoma DBT-9 cells and baby hamster kidney cells stably expressing the MHV receptor (BHK-R) were maintained at 37°C in Dulbecco’s modified Eagle medium (DMEM, Gibco), supplemented with 10% fetal bovine serum (Invitrogen), 100 U/mL penicillin and streptomycin (Gibco), 10 mM HEPES buffer (Corning), and 0.25 µg/mL amphotericin B (Corning). BHK-R cells were also supplemented with 0.8 mg/mL G418 sulfate (Corning). Cells were routinely washed with Dulbecco’s phosphate-buffered saline without calcium chloride or magnesium chloride (PBS−/−). Cells were detached during passage and expansion with 0.05% trypsin-EDTA (Gibco).

### Viruses and amino acid conservation

All work with MHV was performed using the recombinant WT strain MHV-A59 (GenBank accession number AY910861.1). MHV infectious clones were used as templates for mutagenesis and infection experiments (44). Site-directed mutagenesis by “round-the-horn” PCR was used to generate substitutions at the indicated sites (45). MHV infectious clone F fragment was used as a template to substitute nucleotides 18,167–18,169 (nsp14 T3A), 18,179–18,181 (nsp14 K7A), 18,182–18,184 (nsp14 D8A), 18,209–18,211 (nsp14 H17A), 18,305–18,307 (nsp14 T49N), 18,314–18,316 (nsp14 R52Q), and 18,404–18,406 (nsp14 V82I) using the following primers: T3AF (5′-GCCAATTTGTTTAAGGATTGTAGCAGGAG-3′), T3AR (5′- AGTACACTGTAATCGTGGATTGTTAAT-3′); K7AF (5′- GCCGATTGTAGCAGGAGCTATGTAG-3′), K7AR (5′- AAACAAATTTGTAGTACACTGTAATCGT-3′); D8AF (5′- GCATGTAGCAGGAGCTATGTAGG-3′), D8AR (5′- CTTAAACAAATTTGTAGTACACTGTAATCGT); H17AF (5′-GCACCAGCCCATGCAC-3′), H17AR (5′-ATATCCTACATAGCTCCTGC-3′); T49NF (5′- CTATTCGCGGCTTATATCACTC-3′), T49NR (5′-TTGACAGCAGAATCAGCAAC-3′); R52QF (5′-ACTTATATCACTCATGGGATTCAAGC-3′), R52QR (5′-TGCGAATAAGTGACAGCAGAATCAG-3′); V82IF (5′-ACAGAGCCTGGGTTGG-3′), V82IR (5′-TACGTTTGATAGCTTCATCTCT-3′); and K7A/D8AF (5′-GCCGCATGTAGCAGGAGCTATG-3′), K7A/D8AR (5′-AAACAAATTTGTAGTACACTGTAATCGTG-3′). All primers were 5′-phosphorylated with T4 polynucleotide kinase using an ATP-containing reaction buffer (NEB). Template backbone DNA was digested with DpnI (NEB), and amplified DNA was separated by electrophoresis and extracted from agarose (Promega). Ligated DNA was transformed into Top 10 competent *Escherichia coli* cells (Thermo) and amplified in liquid culture, and sequences were confirmed by Sanger sequencing. Assembly and recovery of recombinant MHV have been described previously (44). Electroporated cells were monitored for CPE, and cell flasks were frozen at −80°C when approximately 80% of the monolayer was involved in CPE. Cells were thawed; debris was pelleted; and virus-containing supernatants were aliquoted and stored at −80°C (passage 0). The P0 stocks were used for all experiments above, except for T3A virus, in which case the P1 stocks were used. Engineered mutations of the P0 or P1 stocks used for experiments were confirmed by Sanger sequencing. Samples of viral stock supernatants were collected in TRIzol (Ambion), and viral RNA was extracted by chloroform extraction and purified using the KingFisher MagMAX Viral/Pathogen Nucleic Acid Isolation Kit (Thermo). Viral cDNA was generated with SuperScript IV reverse transcriptase (Thermo) using random hexamers and oligo(dTs). Amplicons (each 3–4 kb in length) were generated via PCR using EasyA polymerase (Agilent) and Sanger sequenced.

### Amino acid sequence conservation

To determine conservation across multiple coronaviruses, multiple sequence alignments were generated using MacVector. The following sequences were used for reference to compare amino acid identity by the NCBI Basic Local Alignment Search Tool: MHV_A549 (YP_ 009915697.1) and SARS-CoV-2 (YP-009725306.1).

### Virus replication assays

DBT-9 cells were plated at a density of 6e5 cells per well 24 h before infection. Cells were then infected at an MOI of 0.01 PFU/cell for 1 h. Inocula were removed, and the cells were washed with PBS before addition of prewarmed medium. Supernatants were harvested at the indicated times post-infection and titers were determined by plaque assay.

### Plaque assays and RT-qPCR

Plaque assays were performed in subconfluent DBT cells seeded in six-well plates. Serial dilutions were plated in duplicate and overlaid with 1% agar in DMEM. Titers were scored at 24 hpi. Genome quantification was determined by one-step RT-qPCR for monolayer-derived RNAs extracted with TRIzol and purified with a KingFisher MagMAX Viral/Pathogen Nucleic Acid Isolation Kit (Thermo) according to the manufacturer’s protocol. Viral RNA was detected on a QuantStudio 3 real-time PCR system (Applied Biosystems) by TaqMan Fast Virus 1-Step Master Mix chemistry (Applied Biosystems) using a 5′ 6-carboxyfluorescein (FAM) and 3′ black hole quencher 1 (BHQ-1)-labeled probe (5′- *TTCTGACAACGGCTACACCCAACG*) and forward (5′- *AGAAGGTTACTGGCAAACTG*) and reverse (5′- *TGTCCACGGCTAAATCAAAC*) primers corresponding to nsp2. RNA copy numbers were determined using an nsp2 RNA standard derived from the MHV A fragment.

### Competitive fitness assay

The MHV competitive fitness assay was previously described in detail (39–41). Briefly, subconfluent DBT-9 cells were coinfected with the indicated virus and a barcoded WT-MHV reference virus with seven silent mutations in nsp2 (1301-CAGCAGT-1307) at a total MOI of 0.1 PFU/cell (0.05 MOI for each virus) in three independent lineages. The resulting virus was passaged four additional times, each at a constant MOI of 0.1 PFU/cell. Viral RNA from each passage supernatant was extracted in TRIzol and purified with a KingFisher II (Thermo Fisher Scientific) according to the manufacturer’s protocol. RNA corresponding to the barcoded WT reference and test viruses was determined by one-step RT-qPCR using SYBR green. BC WT reference RNA was detected with forward (5′-*CTATGCTGTATACGGACAGCAGT*) and reverse (5′-*GGTGTCACCACAACAATCCAC*) primers, and test virus RNA was detected with forward (5′-*CTATGCTGTATACGGATTCGTCC*-3′) and reverse (5′-*GGTGTCACCACAACAATCCAC*) primers using a Power SYBR green RNA-to-Ct 1-step kit (Applied Biosystems) on a QuantStudio 3 real-time PCR system (Applied Biosystems). The log-transformed cycle threshold (*C_T_*) ratio of test versus reference was plotted over passage. Linear regression of the individual log-transformed *C*_*T*_ plots was used to determine the slopes of linear regression for each lineage. Relative fitness was determined by calculating 10^Slope^ for each lineage, and the relative fitness values were then normalized to the mean of the WT versus WT BC control.

### Nucleoside analog sensitivity studies

For 5-fluorouracil sensitivity assays, DBT-9 cells were preincubated with the indicated concentration of 5-fluorouracil or dimethylsulfoxide (DMSO) control for 30 minutes. Subconfluent monolayers of DBT-9 cells were infected with MHV at an MOI of 0.01 PFU per cell for 1 h at 37°C. The inoculum was removed and replaced with medium containing the indicated compound concentration (5-fluorouracil [Sigma] or EIDD-1931 [MedChemExpress]). Cell supernatants were harvested 24 h post-infection. Titers were determined by plaque assay as previously described.

### Passaging nsp14 mutant viruses

WT MHV, MHV with the nsp14 K7A substitution, and MHV with the nsp14 D8A substitution were each passaged in T25 flasks, and infection was initiated 16 h after plating using 1 mL of passage 0 stock virus. Virus supernatants were harvested at 8 hpi and stored on ice overnight at 4°C, and total RNA from virus-infected monolayers was harvested using TRIzol (Invitrogen). A constant volume of 1 mL was used to initiate subsequent passages for a total of 15 passages.

### Illumina RNA sequencing of viral RNA, processing, and alignment

Total RNA was extracted from monolayers infected with WT and mutant MHV viruses at P1 and P15 using TRIzol (Invitrogen) according to the manufacturer’s instructions. For RNA-seq, total RNA underwent poly(A) selection followed by NovaSeq PE150 sequencing (Illumina) at 15 million reads per sample at the Vanderbilt University Medical Center core facility, Vanderbilt Technologies for Advanced Genomics (VANTAGE). VANTAGE performed base-calling and read demultiplexing. The CoVariant pipeline (46) was used for variant analysis. The first module trims and aligns raw FASTQ files to the viral genome for each specified sample using a standard Bash shell script. To summarize, raw reads were processed by first removing the Illumina TruSeq adapter using Trimmomatic. Reads shorter than 36 bp were removed, and low-quality bases (*Q* score of <30) were trimmed from read ends. The raw FASTQ files were aligned to the MHV-A59 genome (AY910861.1) by using the CoVariant Python3 script command line parameters. For variant analysis, the sequence alignment map (SAM) file was processed using the Samtools suite, and alignment statistics output was generated by Samtools idxstats to an output text file. Nucleotide depth at each position was calculated from the SAM files using BBMap (Bushnell) pileup.sh.

### nsp10/nsp14 fusion cloning protein expression

A MHV (NP_045299) nsp10/nsp14 fusion containing a 2× GGS linker between nsp10 and nsp14 was codon optimized for expression in *E. coli* using IDT codon optimization tool and cloned into a pK27 vector that contains an N-terminal 6x-His-tag, Flag-tag, and a SUMO-tag as originally described for SARS-CoV-2 (42). Plasmids were transformed into C41 (DE3) pLysS competent cells (Sigma-Aldrich CMC0018). Cultures were grown in terrific broth with 50 µg/mL kanamycin at 37°C until they reached an OD600 between 0.8 and 1.0, and then cooled at 4°C for 1 h. Cultures were induced with 0.5 mM isopropyl β-D-1-thiogalactopyranoside (IPTG) and incubated overnight at 18*°*C. The following day, cells were centrifuged, resuspended in 25 mL of lysis buffer (50 mM NaH_2_PO_4_, 300 mM NaCl, 10 mM imidazole, pH 8, complete EDTA-free protease inhibitor tablet from Sigma Aldrich (04693132001)), lysed with a sonicator, recentrifuged at 18,000 × *g* for 30 minutes at 4°C, and the supernatant was filtered through a 0.45 μm filter. After filtering, the supernatant was resuspended 1:5 with lysis buffer and incubated at 4°C with Takara’s Talon affinity resin. Resin was then collected via gravity chromatography and eluted with elution buffer (50 mM NaH_2_PO_4_, 300 mN NaCl, and 500 mM imidazole pH 8). The protein was then dialyzed twice against PBS to remove traces of imidazole and concentrated in a 50K molecular weight PES concentrator (Thermo Scientific, 88541). Protein quantitation was carried out using Pierce Rapid Gold BCA Protein assay (Thermo Scientific, A53225) and a 4%–20% mini-Protean TGX Stain-Free gel (Bio-Rad) was run to confirm protein concentrations. Gel was imaged using ChemiDoc MP Imaging system using stain-free settings.

### FRET-based *in vitro* exonuclease assay

Exonuclease activity was quantified using purified WT and mutant nsp10/nsp14 protein diluted in NSP buffer (50 mM Tris-HCl, 0.0001% [vol/vol] Tween 20, 5% [vol/vol] glycerol, 1.5 mM MgCl_2_, 20 mM NaCl, 0.5 mM tris(2-carboxyethyl)phosphine [TCEP], 0.1 μg/mL bovine serum albumin [BSA]). The affinity tags were not cleaved from the proteins. WT protein (500 nM) was mixed with 250 nM of annealed RNA oligos containing fluor and quencher 5′TexRd-XN/rArCrArArArArCrGrGrCrCrCrA and rA*rA*rA*rU*rA*rG*rG*rG*rC*rC*rG*rU*rU*rU*rU*rG*rU*/3′IAbRQSp/ (*indicates phosphothioate bond). Oligos were diluted then annealed in NSP buffer starting at 95°C for 10 minutes, then stepping down by 5°C every minute thereafter until a final 5 minute step at 25°C. The reaction took place at room temperature, and fluorescence was measured using a Thermo Scientific Varioskan Lux 3020–81011 plate reader iii using (excitation: 590 nm, emission 615 nm) for 30 minutes. For interface and adaptive mutants, protein was normalized to WT concentrations using a combination of BCA assay results and intensity of protein band imaged in a mini-Protean TGX Stain-Free gel. Fluorescence measurements for the first 479 s were used to calculate AUC in GraphPad Prism, and total peak area is reported. For baseline measurements, an average of the first five timepoints of the Probe only control were used.

### Model building of MHV nsp14

A *de novo* model of the MHV nsp14 was generated by ColabFold using the MHV_A59 sequence (YP_009915687.1). The results were then visualized and aligned to the SARS-CoV-2 nsp14 from the solved structure 7N0B in PyMOL (Schrödinger).

### Statistics

Statistical tests were performed using GraphPad (La Jolla, CA) Prism 10 software as described in the respective figure legends.

## Data Availability

FASTQ files for the RNA sequencing variant analysis have been deposited in the National Center for Biotechnology Information Sequence Read Archive under the accession number PRJNA1151693.
